# Effects of Calcium and Manganese on Sporulation of *Bacillus* Species Involved in Food Poisoning and Spoilage

**DOI:** 10.3390/foods8040119

**Published:** 2019-04-07

**Authors:** Martti Tapani Sinnelä, Young Kyoung Park, Jae Hoan Lee, KwangCheol Casey Jeong, Young-Wan Kim, Han-Joon Hwang, Jae-Hyung Mah

**Affiliations:** 1 Department of Food and Biotechnology, Korea University, 2511 Sejong-ro, Sejong 30019, Korea; msinnela@gmail.com (M.T.S.); eskimo@korea.ac.kr (Y.K.P.); jae-lee@korea.ac.kr (J.H.L.); ywankim@korea.ac.kr (Y.-W.K.); hjhwang@korea.ac.kr (H.-J.H.); 2 Department of Animal Sciences, University of Florida, Gainesville, FL 32611, USA; kcjeong@ufl.edu; 3 Emerging Pathogens Institute, University of Florida, Gainesville, FL 32611, USA

**Keywords:** *Bacillus* species, calcium, manganese, mineral, sporulation

## Abstract

Spores are resistant against many extreme conditions including the disinfection and sterilization methods used in the food industry. Selective prevention of sporulation of *Bacillus* species is an ongoing challenge for food scientists and fermentation technologists. This study was conducted to evaluate the effects of single and combined supplementation of calcium and manganese on sporulation of common pathogenic and food spoilage *Bacillus* species: *B*. *cereus*, *B*. *licheniformis*, *B*. *subtilis* and *B*. *coagulans*. Sporulation of *Bacillus* vegetative cells was induced on sporulation media supplemented with diverse concentrations of the minerals. Under the various mineral supplementation conditions, the degree of sporulation was quantified with colonies formed by the *Bacillus* spores. The results revealed that *B*. *licheniformis* and *B*. *cereus* displayed the weakest sporulation capabilities on media with minimal supplementation levels of calcium and manganese. The lowest sporulation of *B*. *subtilis* and *B*. *coagulans* was observed on media supplemented with the highest level of calcium and low levels of manganese. Depending on effect of supplementation on sporulation, the *Bacillus* species were divided into two distinct groups: *B. licheniformis* and *B. cereus*; and *B. subtilis* and *B. coagulans*. The information provides valuable insight to selectively reduce sporulation of *Bacillus* species undesirable in the food industry.

## 1. Introduction

The genus *Bacillus* consists of versatile and ubiquitous gram-positive species that are mostly harmless, however some species demonstrate pathogenic capacities [1,2,3]. Food-borne pathogenic microorganisms are the major causes of food-borne diseases and were reported to be responsible for approximately 600 million cases of food-borne illnesses and 420,000 cases of deaths in 2010 worldwide according to the World Health Organization. While *B. cereus* is known as a common food-borne pathogenic species, other *Bacillus* spp. such as *B. subtilis*, *B. coagulans* and *B. licheniformis* are commonly known as food spoilage bacteria. Although *B*. *licheniformis* and *B*. *subtilis* are known to cause food-borne illnesses and may produce toxins, they are not considered common pathogenic species [3,4,5].

When encountering environmental stresses, the *Bacillus* species may form dormant, metabolically inert spores which are much more resistant than their vegetative counterparts [6,7]. Spores are also found to be resistant against many extreme conditions including the disinfection and sterilization methods used in the food industry. Total elimination or inactivation of bacterial vegetative cells is possible with mild disinfection or sterilization methods, however the same treatment may slightly weaken or even be completely ineffective against spores. Once formed, the complete elimination or inactivation of spores without compromising the sensory qualities of food proves to be an ongoing challenge. The difficulty associated with elimination or inactivation of spores faced in the food industry prompts the need for the preemptive prevention of spores through control of the spore formation process [8,9,10].

Although the sporulation ability of *Bacillus* spp. is mostly determined genetically, environmental conditions such as the mineral composition of media may have a significant effect on sporulation [11,12]. The mineral manganese has been studied extensively in the past, and is known to be essential for both the growth and sporulation of several *Bacillus* spp. [13,14,15]. For that reason, manganese is commonly used in cultivation media to induce sporulation of *Bacillus* spp. [2]. Although calcium supplementation is considered more effective than other minerals at increasing the heat resistance of spores, the effects of calcium on sporulation of *Bacillus* spp. are not well understood [6,16].

Research has already been conducted on several *Bacillus* spp. concerning the effect of manganese on sporulation, and calcium on heat resistance, however the effect of the combined manganese and calcium supplementation on the sporulation of *Bacillus* spp. has not been extensively studied. The objective of this study was to investigate the effects of no, single, and combined supplementation of calcium and manganese on the sporulation of common pathogenic and food spoilage *Bacillus* spp., including *B*. *licheniformis*, *B*. *coagulans*, *B*. *subtilis* and *B*. *cereus*. This is the first study to demonstrate the effects of both single and combined supplementation of calcium and manganese on sporulation of *Bacillus* species involved in food poisoning and spoilage as well as, often, food fermentation.

## 2. Materials and Methods

### 2.1. Microorganisms

The original stocks of *Bacillus* strains were purchased from the Korean Collection for Type Culture (KCTC; Daejeon, Korea), which included *B*. *licheniformis* (KCTC 1918), *B*. *coagulans* (KCTC 3625), *B*. *subtilis* (KCTC 3135) and *B*. *cereus* (KCTC 3624). The strains were grown in Tryptic Soy Broth (TSB; Becton Dickinson, Sparks, MD, USA) for 24 h at 37 °C (for *B*. *licheniformis* and *B*. *coagulans*) or 30 °C (for *B*. *subtilis* and *B*. *cereus*) according to the recommendation of the KCTC, and then stored as glycerol stocks (20%, *v/v*) in a freezer at −20 °C. All the media (and minerals) used in this study were sterilized prior to use.

### 2.2. Preparation of Vegetative Cell Suspension

The stock cultures were activated prior to use by three successive subculturing in TSB for 24 h at the aforementioned temperature specified for vegetative cell growth of each *Bacillus* strain. In the final activation step, 5 mL of the culture of each strain were transferred into 250 mL of TSB and incubated under the same conditions to obtain sufficient numbers of vegetative cells. The cultures were washed three times, and the cell pellets were collected after centrifugation at 15,000× *g* for 5 min at 4 °C. The final concentration of vegetative cell suspension was carefully adjusted to 10^9^ CFU/mL with M/15 Sörensen’s phosphate buffer (disodium hydrogen phosphate—Na_2_HPO_4_ 5.675 g, potassium dihydrogen phosphate—KH_2_PO_4_ 3.63 g in 1 L of distilled water, pH 7.0; chemicals from Sigma-Aldrich Co., St. Louis, MO, USA).

### 2.3. Preparation of Sporulation Media with Cations

Sporulation media supplemented with varying concentrations of calcium and/or manganese divalent cations were prepared using nutrient agar containing 13 g/L of nutrient broth (MB cell, Seoul, Korea) and 20 g/L of agar (Ventech Bio Co., Ltd., Eumseong, Korea). The concentrations of supplemented divalent cations were based on reports by Amaha and Ordal [17], Levinson and Hyatt [18] and Bai [19] with minor modifications. The minerals, CaCl_2_·2H_2_O (calcium chloride dihydrate; Dushefa Biochemie, Harleem, the Netherlands) and MnSO_4_·H_2_O (manganese sulfate monohydrate; Biosesang, Seoul, Korea), were used to supply the cations at concentrations of 0.00, 0.25, 0.50, 1.00 and 2.00 mM and 0.00, 0.10, 0.25 and 0.50 mM, respectively. The media without mineral supplementation were used for control treatments.

### 2.4. Induction of Sporulation

Induction of sporulation was carried out based on a previous study [19]. For sporulation of *Bacillus* vegetative cells, 0.1 mL of vegetative cell suspension was spread onto the aforementioned sporulation media. Spore formation was induced by incubation of vegetative cells for 2 days at the temperature specified for each *Bacillus* strain. To harvest the spores, the agar surface was flooded with 7 mL of sterile M/15 Sörensen’s phosphate buffer and scraped with a disposable plastic cell scraper. The spores obtained from 20 plates were collected together, washed three times with M/15 Sörensen’s phosphate buffer by centrifugation at 19,000× *g* for 10 min at 4 °C, resuspended in 3 mL of the same buffer, and kept in a refrigerator (4 °C) until use.

### 2.5. Enumeration of Spores

To enumerate the spores, 0.1 mL of spore suspension was initially mixed with 0.9 mL of M/15 Sörensen’s phosphate buffer and placed in an 80 °C water bath (Sejong Scientific Co., Bucheon, Korea) for 15 min; heat-shock treatment. Immediately after heat shock, the mixed suspension was cooled in a crushed ice water bath. The treated suspension was serially diluted in sterile 0.1% peptone water, and 0.1 mL of each dilution was spread onto Plate Count Agar (PCA; Becton Dickinson) in duplicate. The colonies were manually counted after incubation under the aforementioned conditions for each *Bacillus* strain.

### 2.6. Statistical Analysis

The experiments were carried out in triplicate, and the data were presented as means and standard deviations of three independent experiments. The significance of differences was determined by analysis of variance (ANOVA) with Fisher’s multiple comparison module of the Minitab statistical software, version 17 (Minitab Inc., State College, PA, USA), and differences with *p*-values of <0.05 were considered statistically significant.

## 3. Results and Discussion

### 3.1. Sporulation Profiles of B. licheniformis and B. cereus

Extensive studies of manganese show that the mineral is essential for both the growth and sporulation of several *Bacillus* spp. [13,14,15]. Calcium supplementation in sporulation media is often associated with increased heat resistance [6,16]. Though supplementation of the sporulation media with both Ca^2+^ (calcium ion) and Mn^2+^ (manganese ion) has been speculated to increase the sporulation capability of *Bacillus* spp., the results obtained in the present study revealed that the response to the presence of the metallic ions varies widely among *Bacillus* species. 

**Figure 1:**
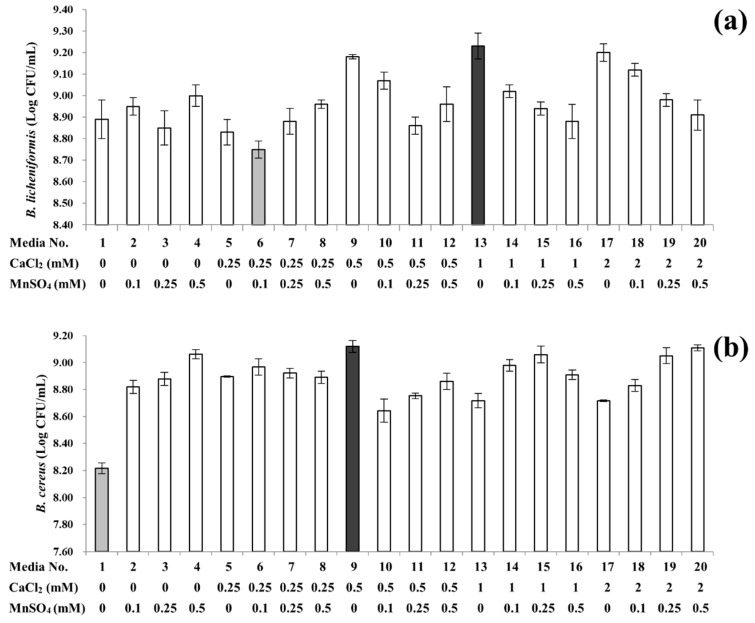
Sporulation profiles of *Bacillus licheniformis* (**a**) and *B*. *cereus* (**b**) under 20 different supplementation conditions. The light grey and dark grey columns highlight the lowest and highest spore yields, respectively. Error bars indicate standard deviations calculated from triplicate experiments.

As shown in Figure 1a and Table S1, *B*. *licheniformis* produced the lowest quantity of spores (8.75 ± 0.04 Log CFU/mL) on media supplemented with the lowest levels of calcium (0.25 mM) and manganese (0.10 mM). In Figure 1b and Table S1, *B*. *cereus* displayed the weakest sporulation capability (8.22 ± 0.04 Log CFU/mL) when grown on the non-supplemented media. Optimum sporulation of *B. licheniformis* (9.24 ± 0.06 Log CFU/mL) was observed on media supplemented solely with 1.00 mM of calcium, while *B*. *cereus* displayed the highest sporulation capability (9.12 ± 0.04 Log CFU/mL) on media supplemented only with 0.50 mM of calcium. On media supplemented solely with a single mineral, the results also indicated that manganese supplementation was less effective than calcium at inducing sporulation, when calcium was supplemented at moderate levels (at least 0.50 mM for *B. licheniformis* and 0.50 mM for *B. cereus*). Interestingly, when manganese was supplemented along with lower levels of calcium, sporulation capabilities were slightly enhanced for both species.

Though *B*. *licheniformis* and *B. cereus* were similarly influenced by calcium and manganese supplementation concentrations as described above, the extent to which supplementations affect sporulation was somewhat different between the two species. In the case of *B*. *licheniformis*, higher levels of calcium supplementation were found to effectively enhance sporulation. The three highest *B*. *licheniformis* spore yields of 9.24 ± 0.06, 9.18 ± 0.01 and 9.15 ± 0.10 Log CFU/mL were obtained on media supplemented solely with calcium at concentrations of 1.00, 0.50 and 2.00 mM, respectively. Direct correlations were not found for media supplemented exclusively with manganese. The non-supplemented media yielded 8.89 ± 0.09 Log CFU/mL of spores, whereas media supplemented solely with manganese did not significantly affect sporulation capabilities (approximately 8.85 to 8.95 Log CFU/mL), regardless of concentration. However, alongside 1.00 or 2.00 mM of calcium, manganese was found to slightly inhibit the sporulation of *B. licheniformis*. In the case of *B*. *cereus*, supplementation of calcium and/or manganese significantly enhanced sporulation. Supplementations at all levels increased the spore yield by approximately 0.4 to 0.9 Log CFU/mL when compared to the spore yield obtained on the non-supplemented media. Furthermore, as the *B. cereus* revealed higher spore yields on most media supplemented with manganese, manganese appeared to be more effective than calcium for sporulation. Similarly, Ryu et al. [7] stated that in the absence of calcium, supplementation of manganese enhanced the spore formation of *B*. *cereus* strains grown in TSB. Nonetheless, it is noteworthy that in the present study, the highest spore yield of *B*. *cereus* was obtained on media supplemented only with calcium at a concentration of 0.50 mM.

### 3.2. Sporulation Profiles of B. subtilis and B. coagulans

**Figure 2:**
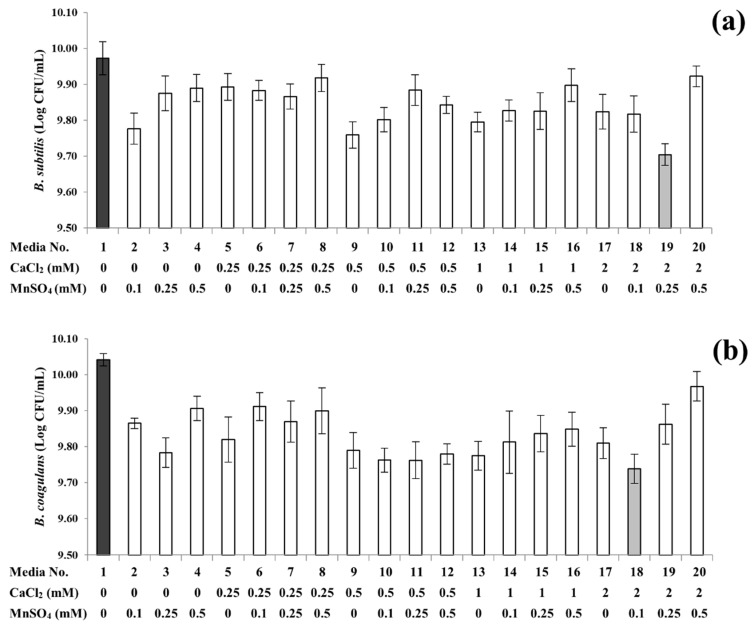
Sporulation profiles of *B*. *subtilis* (**a**) and *B*. *coagulans* (**b**) under 20 different supplementation conditions. The light grey and dark grey columns highlight the lowest and highest spore yields, respectively. Error bars indicate standard deviations calculated from triplicate experiments.

In contrast to *B*. *licheniformis* and *B. cereus*, both *B*. *subtilis* and *B*. *coagulans* displayed the highest sporulation capabilities (9.97 ± 0.05 and 10.04 ± 0.02 Log CFU/mL, respectively) on non-supplemented media and the lowest capabilities (9.70 ± 0.03 and 9.74 ± 0.07 Log CFU/mL, respectively) with 2.00 mM calcium supplementation, as shown in Figure 2a,b and Table S2. On media supplemented solely with a single mineral, the results also indicated that calcium supplementation was less effective than manganese at inducing sporulation, when manganese was supplemented at a moderate level (0.25 mM or more for *B. subtilis* and 0.10 or 0.50 mM for *B. coagulans*). Other studies have also shown that manganese supplementation may enhance the spore formation capabilities of *B*. *subtilis* [13,20] and *B. coagulans* [21]. Conversely, Oomes et al. [22] reported that the addition of calcium to sporulation media increased the expression of genes involved in *B*. *subtilis* sporulation. Nonetheless, *B. subtilis* exhibited the lowest yield of spore production on media supplemented with both the highest concentration of calcium (2.00 mM) and a moderate level of manganese (0.25 mM) in the present study. *B. coagulans* also displayed the lowest yield of spores when grown on media with both the highest amount of calcium supplementation (2.00 mM) and a low level of manganese (0.10 mM). Depending on the concentrations and combinations of supplemented minerals, the spore yields of *B*. *subtilis* were consequently reduced by approximately 0.1 to 0.4 Log CFU/mL. Likewise, the supplementations decreased the spore yields of *B*. *coagulans* by approximately 0.1 to 0.3 Log CFU/mL. When calcium and manganese were not supplemented, both *Bacillus* spp. displayed the highest sporulation capabilities, as described above. The differences in results between previous reports and the present study are likely due to the variances of strains, media and experimental methodologies utilized. Furthermore, when compared to the single mineral supplementations, the results also imply that undetermined interactions between calcium and manganese may exist when supplemented together. Therefore, further studies on the complex interactions are necessary to understand the underlying mode(s) of action of combined supplementation on sporulation. It is worth reiterating that on media supplemented with a single mineral, manganese was favorable for the sporulation of *B*. *subtilis* and *B*. *coagulans*, as spore yields on media supplemented solely with manganese were relatively higher than the exclusive supplementation of calcium. Similar to *B. cereus*, the results for *B. subtilis* and *B. coagulans* highlighted the importance of manganese for sporulation in many *Bacillus* spp., as proposed in other studies [13,14,15].

All things considered, depending on effect of supplementation on sporulation, the *Bacillus* species were divided into two distinct groups: *B*. *licheniformis* and *B*. *cereus*; *B*. *subtilis* and *B*. *coagulans*. Contrary to the sporulation patterns of the two groups observed in the present study, the complete genomic sequence of *B*. *licheniformis* has been found to be the more similar to *B*. *subtilis* than other *Bacillus* species [23,24]. It is therefore necessary to compare the expression of genes responsible for sporulation among species of *Bacillus* through further research.

### 3.3. Further Implications

*Bacillus* spp. are often regarded as contaminants in many foods [3,4,5], including fermented soybeans [25]. Many types of fermented soybean foods are typically prepared with salt at concentrations up to 10%, but may reach 20% or higher. Upon consideration of previous studies [26,27,28], it appears that under saline conditions either formation of spores or prevention of spore germination may occur through low water activity. Therefore, to ensure the quality and safety of food products, it is often imperative to eliminate or reduce spores rather than vegetative cells. Dormant *Bacillus* spores are extremely resistant to environmental stresses, enabling the spores to survive the wide variety of sterilization treatments used in the food industry [29,30]. Thus, development of methods to effectively prevent sporulation of *Bacillus* spp. may help to minimize contamination, ensuring the quality and safety of food products.

The species tested in the present study (*B. subtilis*, *B*. *coagulans* and *B. licheniformis*) are known to be the major contributors to the fermentation of soybeans (at least in some cases) as well as spoilage microorganisms in fermented soybeans [31,32]. Finely controlling the sporulation of different *Bacillus* species may facilitate the selective elimination of undesirable species (especially, *B. cereus*), while preserving beneficial *Bacillus* species in food products. The results found in this study may contribute to a better understanding of differences in sporulation between the two groups of *Bacillus* species, which may in turn be helpful in at least partially reducing contamination of fermented soybeans and other foods by undesirable *Bacillus* spores. Selective prevention of sporulation of undesirable *Bacillus* species may be more satisfactorily achieved if the knowledge obtained in this study is combined with other intervention strategies, which remains to be further studied. The intervention strategies may involve the use of heat-resistant spores as protective cultures, adjustment of conditions used for heat treatment and storage, and use of proper minerals and other additives affecting sporulation and heat resistance, which have been intensively studied with *Clostridium sporogenes* [33,34,35,36].

## 4. Conclusions

This study was conducted to analyze the effect of calcium and manganese on the sporulation of four common pathogenic and food spoilage *Bacillus* species: *B. licheniformis*, *B*. *cereus*, *B*. *coagulans* and *B*. *subtilis*. The results demonstrated that the lowest sporulation capacities of *B. licheniformis* and *B. cereus* were observed on media supplemented in minimal concentrations of minerals and non-supplemented media, respectively. Interestingly, the highest sporulation of *B. subtilis* and *B. coagulans* was observed on media without mineral supplementation. Moreover, the highest sporulation yields of *B*. *licheniformis* and *B*. *cereus* were obtained on media supplemented solely with calcium, while the lowest yields of *B*. *subtilis* and *B*. *coagulans* spores were obtained on media with high levels of calcium supplementation. Based on the observed spore yields, the tested *Bacillus* species may be divided into two groups: *B*. *licheniformis* and *B*. *cereus*; and *B*. *subtilis* and *B*. *coagulans*. This study may provide insight in the dynamics of sporulation useful for developing intervention and risk assessment strategies to ensure the quality and safety of food products. This study also suggests that selective prevention of sporulation of undesirable *Bacillus* species is possible, while not or less affecting beneficial *Bacillus* species in food products.

## Supplementary Materials

The followings are available online at https://www.mdpi.com/2304-8158/8/4/119/s1, Table S1: Statistical comparison of sporulation of *B. licheniformis* and *B. cereus* influenced by calcium and manganese supplementation concentrations, Table S2: Statistical comparison of sporulation of *B. subtilis* and *B. coagulans* influenced by calcium and manganese supplementation concentrations.
