# Supplementary material for: Effects of Calcium and Manganese on Sporulation of Bacillus Species Involved in Food Poisoning and Spoilage
Source: Foods. 2019 Apr 7;8(4):119. doi: 10.3390/foods8040119 (PMC6517867; doi:10.3390/foods8040119)
Supplement: Supplementary file 1 [file foods-08-00119-s001.pdf]

Table S1. Statistical comparison of sporulation of *B. licheniformis* and *B. cereus* influenced by calcium and manganese supplementation concentrations.

Calcium (mM)	Manganese (mM)	Viable spore count (Log CFU/mL) [1]	
		B. licheniformis	***B. cereus***
0.00	0.00	8.89 ± 0.09 ^{HIJKL}	8.22 ± 0.04 $^{\text{K},*}$
	0.10	8.95 ± 0.04 ^{FGHIJ}	8.80 ± 0.07 ^{I}
	0.25	8.85 ± 0.08 ^{KL}	8.84 ± 0.10 ^{GHI}
	0.50	8.94 ± 0.10 ^{FGHIJ}	9.02 ± 0.07 ^{BCD}
0.25	0.00	8.83 ± 0.06 ^{LM}	8.90 ± 0.05 ^{EFGH}
	0.10	8.75 ± 0.04 $^{\text{M},*}$	8.97 ± 0.09 ^{CDEF}
	0.25	8.88 ± 0.06 ^{IJKL}	8.93 ± 0.03 ^{DEFG}
	0.50	8.96 ± 0.02 ^{FGH}	8.89 ± 0.05 ^{EFGHI}
0.50	0.00	9.18 ± 0.01 ^{AB}	9.12 ± 0.04 $^{\text{A},+}$
	0.10	9.07 ± 0.04 ^{CDE}	8.65 ± 0.13 ^{J}
	0.25	8.86 ± 0.04 ^{L}	8.65 ± 0.12 ^{J}
	0.50	8.96 ± 0.08 ^{FGHIJ}	8.89 ± 0.09 ^{EFGHI}
1.00	0.00	9.24 ± 0.06 $^{\text{A},+}$	8.69 ± 0.09 ^{J}
	0.10	9.02 ± 0.03 ^{DEF}	8.98 ± 0.06 ^{CDEF}
	0.25	8.94 ± 0.03 ^{FGHIJK}	8.99 ± 0.12 ^{CDE}
	0.50	8.88 ± 0.08 ^{JKL}	8.89 ± 0.06 ^{FGHI}
2.00	0.00	9.15 ± 0.10 ^{BC}	8.69 ± 0.14 ^{J}
	0.10	9.07 ± 0.10 ^{CD}	8.81 ± 0.06 ^{HI}
	0.25	8.98 ± 0.04 ^{EFG}	9.05 ± 0.06 ^{ABC}
	0.50	8.91 ± 0.07 ^{GHIJKL}	9.11 ± 0.10 ^{AB}
Average		8.97 ± 0.12	8.85 ± 0.21

[1] Mean ± standard deviation calculated from triplicates; $^{\text{A-M}}$ mean values in the same column that are followed by different letters are significantly different ($p < 0.05$); * the lowest viable spore count obtained; $^{+}$ the highest viable spore count obtained.

Table S2. Statistical comparison of sporulation of *B. subtilis* and *B. coagulans* influenced by calcium and manganese supplementation concentrations.

Calcium (mM)	Manganese (mM)	Viable spore count (Log CFU/mL) [1]	
		B. subtilis	***B. coagulans***
0.00	0.00	9.97 ± 0.05 [A,+]	10.04 ± 0.02 [A,+]
	0.10	9.78 ± 0.04 [FG]	9.86 ± 0.01 [CDEF]
	0.25	9.87 ± 0.05 [BCD]	9.78 ± 0.05 [GHIJ]
	0.50	9.89 ± 0.04 [BC]	9.91 ± 0.03 [CD]
0.25	0.00	9.89 ± 0.04 [BC]	9.82 ± 0.06 [EFGHI]
	0.10	9.88 ± 0.03 [BC]	9.91 ± 0.04 [BC]
	0.25	9.87 ± 0.03 [CD]	9.86 ± 0.10 [CDEFGH]
	0.50	9.92 ± 0.04 [AB]	9.90 ± 0.06 [CD]
0.50	0.00	9.76 ± 0.04 [G]	9.79 ± 0.05 [HIJ]
	0.10	9.80 ± 0.03 [EFG]	9.76 ± 0.03 [IJ]
	0.25	9.88 ± 0.04 [BC]	9.76 ± 0.05 [IJ]
	0.50	9.84 ± 0.02 [CDE]	9.79 ± 0.01 [FGHIJ]
1.00	0.00	9.80 ± 0.03 [EFG]	9.80 ± 0.05 [EFGHIJ]
	0.10	9.83 ± 0.03 [DEF]	9.87 ± 0.02 [CDE]
	0.25	9.83 ± 0.05 [DEF]	9.87 ± 0.02 [CDEF]
	0.50	9.90 ± 0.05 [BC]	9.85 ± 0.05 [DEF]
2.00	0.00	9.82 ± 0.05 [DEF]	9.81 ± 0.04 [EFGHI]
	0.10	9.82 ± 0.05 [EF]	9.74 ± 0.07 [J,*]
	0.25	9.70 ± 0.03 [H,*]	9.86 ± 0.06 [CDEF]
	0.50	9.92 ± 0.03 [AB]	9.97 ± 0.04 [B]
Average		9.85 ± 0.06	9.85 ± 0.07

[1] Mean ± standard deviation calculated from triplicates; [A-J] mean values in the same column that are followed by different letters are significantly different ($p < 0.05$); * the lowest viable spore count obtained; [+] the highest viable spore count obtained.
